# Correlations of Self-Reported Androgen Deficiency in Ageing Males (ADAM) with Stress and Sleep among Young Adult Males

**DOI:** 10.3390/healthcare6040121

**Published:** 2018-10-01

**Authors:** Camille M. Charlier, Makenzie L. Barr, Sarah E. Colby, Geoffrey W. Greene, Melissa D. Olfert

**Affiliations:** 1 Clinical & Translational Science, Health Sciences Center, West Virginia University, Morgantown, WV 26506, USA; ccharlie@hsc.wvu.edu; 2 Davis College of Agriculture, Natural Resources and Design, Department of Animal & Nutritional Sciences, Agricultural Science Building, G025, West Virginia University, Morgantown, WV 26506, USA; mbarr6@mix.wvu.edu; 3 Department of Nutrition, University of Tennessee, 1215 W Cumberland Ave, 229 Jesse Harris Building, Knoxville, TN 37996, USA; scolby@utk.edu; 4 Department of Nutrition and Food Sciences, University of Rhode Island, Kingston, RI 02881, USA; ggreene@uri.edu

**Keywords:** androgen deficiency, ADAM score, low testosterone, young adult males, stress, sleep

## Abstract

Androgen deficiency in males has traditionally been predominantly limited to older men aged 50+ years. However, little is known of the correlation between hormonal disruption, stress, and sleep in college-aged males. This cross-sectional study investigates lifestyle behavior patterns in young men and a screening for potential androgen deficiency. A survey of 409 male students, as part of a larger USDA-funded GetFruved study, was analyzed for this subproject. Survey instruments used include the Androgen Deficiency in the Aging Male Questionnaire (ADAM) to assess for inadequate ADAM scores, the Perceived Stress Scale to measure stress levels and the Pittsburgh Sleep Quality Index to evaluate sleep quality. In total, 144 male participants (35%) met criteria for potential androgen deficiency defined by the ADAM questionnaire. Correlation was found between having a positive ADAM score and both increased stress levels (*p* < 0.001) and poor sleep quality (*p* < 0.001), with stress displaying the strongest effect (*p* < 0.001 vs *p* = 0.124). An increased prevalence of having a positive ADAM score versus established norms for this age group was also noted. These findings highlight the need for investigation of endocrine disruptions in young men.

## 1. Introduction

It is known that male androgen deficiency is more predominant among older-men, with prevalence rates of 34% for men in their 60s, 91% for men in their 80s, and less than 5% in males aged 20–29 [1]. Note that, due to testosterone being the primary male androgen hormone, the term male androgen deficiency, hypogonadism, male low testosterone, and male testosterone deficiency are used interchangeably in the literature. Additionally, hormone-based treatment is typically referred to as testosterone treatment for all of these conditions. However, the widespread rise of obesity, and its accompanying host of co-morbidities, including type 2 diabetes (T2DM), cardiovascular disease (CVD), and metabolic syndrome (MetS), is changing long-held assumptions about hormonal disruptions. Male hypogonadism has been found to positively correlate with all three of these co-morbid conditions [2,3,4,5,6]. Although prevalence rates are difficult to assess for individuals with CVD and MetS, in males with T2DM, hypogonadism prevalence is estimated to be 33–50% [7]. These trends are particularly concerning in younger demographics which have experienced significant increases in obesity rates in the past fifteen years. The Centers for Disease Control (CDC) reported that prevalence of obesity among American teenagers almost doubled from 2001 to 2015, going from 10.5% to 20% [8]. Additionally, nearly a third of young adults in the U.S. were considered obese in 2015 [8]. Interestingly, although treatment of hypogonadism remains challenging [9], medical management of androgen deficiency in males has displayed encouraging results. In obese men with low testosterone, testosterone replacement resulted in improved body composition and reduced waist circumference, leading to sustained weight loss and reduced body mass index (BMI) [10]. In male patients with androgen deficiency and T2DM, a disorder itself with a high prevalence of male hypogonadism [11,12], similar treatment not only yielded decreased subcutaneous fat and increased lean mass, it also improved insulin sensitivity [13].

In addition to specific medical diagnoses, certain lifestyle factors with well-established hormonal components can significantly affect health. Sleep and stress have been found to both be mediated by hormones and to have the capacity to alter normal endocrine function [14,15]. Indeed, male testosterone production appears to be linked to sleep cycles, including REM sleep [16]. Furthermore, young adults frequently experience poor sleep, with only approximately a third of college students reporting good sleep quality using the Pittsburgh Sleep Quality Index (PSQI) scale and 29.4% reporting getting the ≥8 h of sleep recommended for this population [17]. Emerging evidence suggests that reduced sleep is a potential risk factor for obesity [18]. Stress (personal and academic) has been shown to significantly affect sleep quality in young adults [17]. Additionally, male teenagers with decreased stress resilience, or reduced ability to successfully cope with an issue, have been shown to be at increased risk of developing T2DM in adulthood, even when accounting for traditional risk factors such as BMI and family history [19]. Endocrine disruptions can result in a variety of physiological, cognitive and emotional conditions, which can each bring their own negative contribution to overall health, resulting in a snowballing effect damaging long-term quality of life and leading to increased healthcare costs.

The relationships among having a potential androgen deficiency, sleep, and stress in young adult males are unknown. This study investigated 409 male college students enrolled in the USDA-funded GetFruved study to examine if potential androgen deficiency, sleep, and stress are related via self-reported questionnaires.

## 2. Materials and Methods

Over 1200 college students across eight U.S. universities were surveyed as a part of a USDA-funded GetFruved study, a project aimed at identifying and improving lifestyle behaviors and environments associated with obesity prevention on university campuses. Male students (n = 409) were analyzed as a part of this subproject. Approval to use the dataset was granted by the University of Tennessee Institutional Review Board prior to study implementation. This material is based upon work that is supported by the National Institute of Food and Agriculture, U.S. Department of Agriculture, under award number 2014-67001-21851.

The validated survey instrument used included the Androgen Deficiency in the Aging Male Questionnaire (ADAM) to assess potential androgen deficiency [20,21]. This tool has been utilized to assess low bioavailable testosterone in older males aged 40–62 years and evaluated the change of these scores after administration of testosterone to those found to be low [21]. The ADAM tool was analyzed to have an 88% sensitivity and 60% specificity in capturing those males with lower testosterone. Specifically, 18 of the 21 men given testosterone treatment found improvement in their ADAM scores. Authors state the ADAM questionnaire poses an acceptable tool for screening tool for capturing males with low bioavailable testosterone.

**Figure 1:**
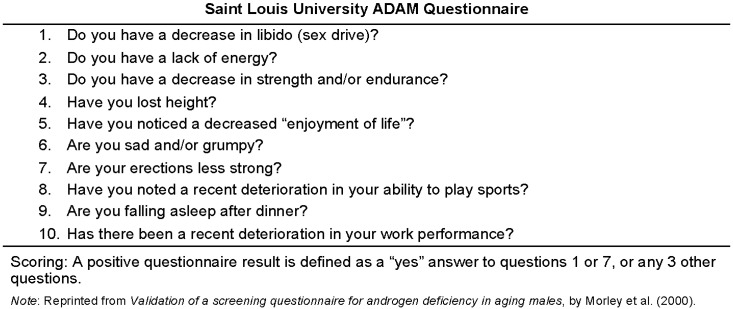
Androgen Deficiency in the Aging Male Questionnaire (ADAM). This non-invasive screening tool was administered to all participants to assess androgen deficiency.

The ADAM questionnaire was scored using the defined instructions listed in Figure 1, with a positive ADAM score being suggestive of a potential androgen deficiency [21]. A positive score was indicated as a “yes” answer to Question 1 or 7, or any other three variables. Per the scoring, the ADAM tool is then placed into a binary variable of “positive” or “negative”. Note that participants with a positive ADAM score are henceforth labeled as “ADAM positive”, with their negative counterparts being referred to as “ADAM negative”.

Additional validated tools to assess these male’s lifestyle traits included Cohen’s 14-item Perceived Stress Scale (PSS) to measure stress levels [22] and the Pittsburgh Sleep Quality Index (PSQI) to evaluate sleep quality [23]. Sleep quality was further categorized per PSQI guidelines, with scores of 0–4 indicating good sleep quality and scores ≥5 indicating poor or inadequate sleep quality [23].

Wilcoxon tests were used on nonparametric data to compare means for the following variables: height, weight, BMI, PSS scores, and PSQI scores. Pearson Chi-Squared test was used to assess the relationship between ADAM scores and PSQI categories. Logistic Regression Analysis and Agreement Analysis of the data were conducted to assess the effect of sleep and stress on ADAM scores. All analyses were completed using JMP [24] and SAS [25] software.

## 3. Results

There were 409 males from eight different states included in analysis (Table 1). Their average height, weight BMI and waist circumference were 176.06 ± 7.40 cm, 77.43 ± 15.98 kg, 24.90 ± 4.62 kg/m^2^, and 82.94 ± 10.49 cm respectively (all Wilcoxon analyses non-significant between ADAM positive and ADAM negative groups; Table 1). The sample was predominantly composed of individuals identifying their race as 70.2% White, 17.1% Hispanic/Latino, 14.4% Asian, 12.0% Black only, 8.8% other (including Alaska Native/American Indian, Native Hawaiian/Pacific Islander and Other) (Table 1).

Overall, males had a PSS of 23.23 ± 7.40 and a PSQI score of 5.72 ± 2.64. Following PSQI categorization, good sleep quality was reported by 145 (36.16%) participants and poor sleep quality by 256 (63.84%) participants.

There were 144 (35.21%) male subjects that met the criteria for having a potential androgen deficiency as defined by positive ADAM questionnaire. Height, weight and BMI were not found to be significantly different between the ADAM positive and ADAM negative participants (Table 2).

**Figure 2:**
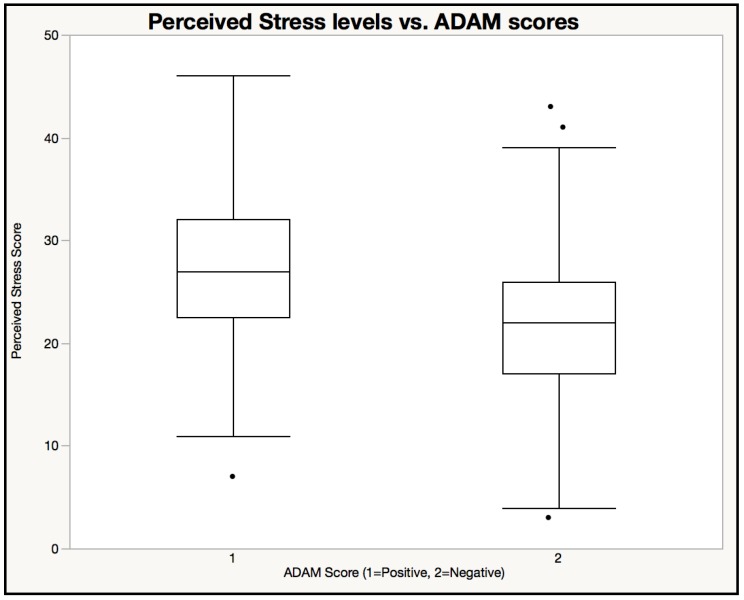
Perceived Stress levels vs. ADAM scores. Participants with positive ADAM scores self-reported higher levels of stress (PSS Mean = 26.82 ± 6.91) than participants with negative ADAM scores (PSS Mean = 21.26 ± 6.91).

Figure 2 showing box plot of PSS among ADAM positive and negative males. Participants with positive ADAM scores self-reported higher levels of stress (PSS Mean = 26.82 ± 6.91) than participants with negative ADAM scores (PSS Mean = 21.26 ± 6.91) (Z = 7.194, *p* < 0.001, Wilcoxon).

**Figure 3:**
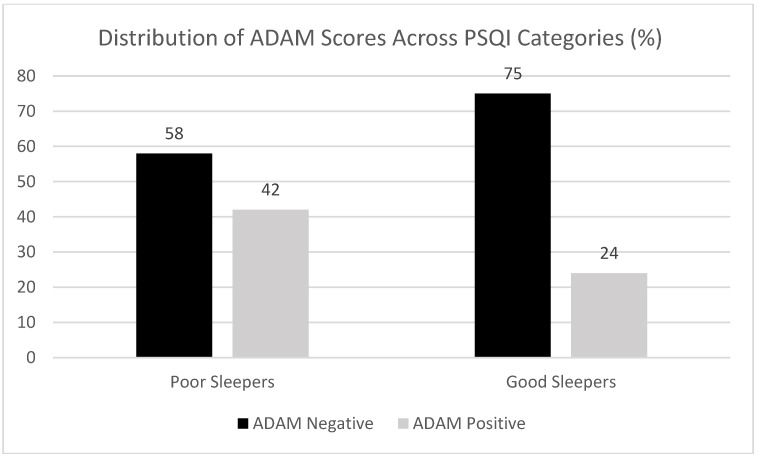
Distribution of ADAM Scores across PSQI Sleep Quality Categories. Participants with positive ADAM scores represented a larger percentage of the sample’s “poor sleeper” group (42%) than it did the “good sleeper” group (24%).

Mean PSQI score of the ADAM positive males was 6.68 ± 2.92 versus 5.19 ± 2.31 for the ADAM negative participants (Z = 5.177, *p* = 2.3 × 10^−7^, Wilcoxon). Sleep findings were consistent following categorization, with poorer sleep quality observed in ADAM positive participants (χ^2^ (1) = 12.621, *p* < 0.001, Pearson Chi-Squared). In ADAM positives, categorized PSQI scores indicated that good sleep quality was reported by 35 (24.65%) participants and poor sleep quality by 107 (75.35%) participants. In ADAM negatives, categorized PSQI scores indicated that good sleep quality was reported by 110 (42.47%) participants and poor sleep quality by 149 (57.53%) participants. Conversely, ADAM positive subjects represented 42% of the “poor sleepers” but only 24% of the “good sleepers”, as shown in Figure 3.

Univariate logistic regression showed statistically significant effects of increased stress (*p* < 0.001) and poor sleep (*p* = 0.0003) on ADAM scores. When modeled together, stress showed a stronger effect than sleep on ADAM scores (χ^2^ (1) = 36.68, *p* < 0.001 versus χ^2^ (1) = 2.37, *p* = 0.1235 respectively).

## 4. Discussion

In young adult men, there was a correlation between a positive ADAM score indicating a potential androgen deficiency and both increased stress levels and poor sleep quality, with stress displaying the strongest effect on ADAM scores. These findings highlight the potential relationships within the testosterone–stress–sleep triad in young adult males. The observed connection between hypogonadism and sleep appears logical given the established role of testosterone in REM sleep cycles [16]. However, the stronger effect of stress than sleep on ADAM results was interesting and potentially suggestive of the co-depending nature of stress and sleep. As stress increases, sleep quality is likely to decrease thereby resulting in sleep as a compounding stressor. Similarly, decreased sleep quality itself may lead to increased stress levels. As such, true distinction between the two variables remains challenging.

Additionally, the high proportion of male subjects scoring positive on the ADAM questionnaire in this study appears to indicate a potentially higher prevalence of hypogonadism in young males than previously reported. Although this sample was a small convenience sample so nationwide prevalence conclusions cannot be made, an ADAM positivity rate of 35.21% was far greater than the estimated 5% androgen deficiency rate in young males [1]. One reason for such a large discrepancy could simply be an underestimation of androgen deficiency prevalence in young men in the current literature. Indeed, up to date estimates are not readily available and research in the field is primarily relying on the 2001 Baltimore Longitudinal Study on Aging. Several factors such as public health changes (i.e., increased obesity) could have reasonably affected the prevalence of androgen deficiency in young adult males over the past sixteen years. Most universities sampled in this study are located in states with high obesity rates. Obesity being linked to hormonal disruptions, this may have played a role in these findings. Indeed, four of the universities represented in this sample come from states ranked in the top ten for highest obesity rates. Furthermore, all but one institutions are located in a state ranked in the top 25 for highest obesity rates [26].

Another limitation of this study was the use of a screening questionnaire to assess for a potential androgen deficiency. By the very nature of its questions, the ADAM questionnaire may lead to a positive result independently of any androgen changes. For example, a patient’s answer to question #2 (“Do you have a lack of energy”) could have multiple potential causes, as could responses to Questions 3, 6, and 10. Furthermore, hormonal dysfunctions can be challenging to pinpoint and even the best screening tools have margins for errors. The ADAM questionnaire was designed for older men as androgen deficiency in males has conventionally predominantly been a concern in males ≥50 years old. This particular screening tool was selected as it is the most widely used one clinically and no young men focused instrument is currently available. However, it has not been specifically validated in younger males, representing a potential shortcoming of this study. Indeed, the ADAM questionnaire may not be valid in younger men. Further validation of the test in this population is strongly warranted. Specifically, when looking into the questions used for the ADAM questionnaire, potential shortening of the tool could be useful. For example, a question regarding loss of height in young adult college students may not be useful as muscle mass and structure tend to not deteriorate in males until after their fifth decade of life that encompasses hormonal deviations and reductions in exercise [27].

In light of our results and current obesity trends, further investigation, including laboratory bloodwork to test testosterone, is recommended: (1) to identify any significant changes to the historical prevalence of androgen deficiency in younger male demographics; and (2) to confirm relationships among clinically diagnosed androgen deficiency, stress, and sleep using physiological markers. Note that literature recommends testing of free or bioavailable testosterone as total testosterone levels can fail to reveal actual testosterone decline, particularly in individuals with milder forms of hypogonadism [28,29]. Unfortunately, with this multi-state sample of young males, we were unable to obtain these biochemical data. Indeed, bioavailable testosterone is the most widely used test in the literature pertaining to the screening for androgen deficiency [21] and should likely be used to ensure both accuracy and consistency [30]. Future studies should utilize this measure of testosterone, especially when validating this tool in a younger population. Overall, this study confirms the need for research to further examine hormonal disruptions and potential health ramifications in young adult males.

## Figures and Tables

**Table 1:** Demographics. Race, and state of the overall sample and the ADAM-positive sub-sample.

Demographic		Frequency (%)	
		Male Sample	ADAM Positive Sub-Sample
		*N* = 409	*N* = 144
Race *	Alaska Native/American Indian	16 (3.91)	5 (3.47)
	Asian	59 (14.43)	21 (14.58)
	Black/African-American	49 (11.98)	18 (12.50)
	Hispanic/Latino	70 (17.11)	21 (14.58)
	Native Hawaiian/Pacific Islander	3 (0.73)	1 (0.69)
	White	287 (70.17)	100 (69.44)
	Other or Choose not to answer	17 (4.16)	8 (5.56)
State	Alabama	23 (5.62)	9 (6.25)
	Florida	79 (19.32)	28 (19.44)
	Kansas	35 (8.26)	11 (7.64)
	Maine	63 (15.40)	14 (9.72)
	New York	65 (15.89)	20 (13.89)
	South Dakota	15 (3.67)	6 (4.17)
	Tennessee	85 (20.78)	33 (22.92)
	West Virginia	40 (9.78)	21 (14.58)
	Choose not to answer	4 (0.98)	2 (1.39)

* Individuals were able to classify themselves as more than one race (i.e. White and Hispanic/Latino) making the total percentage of race > 100%.

**Table 2:** Anthropometrics by ADAM scores. Height, weight, and BMI of the overall sample, including comparison between positive and negative ADAM scores with no statistically significant difference noted.

Anthropometric	Total Male Sample	ADAM SCORES		Significance
		Positive	Negative	
Height (cm)	176.06 ± 7.40	175.50 ± 7.23	176.36 ± 7.48	Z = −0.975, *p* = 0.3293, Wilcoxon
Weight (kg)	77.43 ± 15.98	77.95 ± 16.67	77.15 ± 15.62	Z = 0.323, *p* = 0.7460, Wilcoxon
BMI (kg/m^2^)	24.90 ± 4.62	25.20 ± 4.88	24.74 ± 4.48	Z = 0.905, *p* = 0.3652, Wilcoxon
Waist Circumference (cm)	82.94 ± 10.49	82.46 ± 9.66	83.19 ± 10.92	Z = 0.7542, *p* = 0.7538, Wilcoxon

Wilcoxon test used on nonparametric height, weight, and BMI in relationship to ADAM positive and negative scores.
